# Reading Struggle Stories of Role Models Can Improve Students' Growth Mindsets

**DOI:** 10.3389/fpsyg.2021.747039

**Published:** 2021-10-28

**Authors:** Xu Du, Sheng Yuan, Ying Liu, Xuejun Bai

**Affiliations:** ^1^Faculty of Psychology, Tianjin Normal University, Tianjin, China; ^2^Key Research Base of Humanities and Social Sciences of the Ministry of Education, Academy of Psychology and Behavior, Tianjin Normal University, Tianjin, China; ^3^Tianjin Social Science Laboratory of Students' Mental Development and Learning, Tianjin, China

**Keywords:** growth mindset, struggle story, perseverance, role model, intervention

## Abstract

Previous studies have suggested that reading stories of role models can improve the growth mindsets of students. The current study aimed to investigate the types of stories that can increase the growth mindsets of high school students, undergraduates, and graduates and how many specific stories undergraduates with low and high perseverance need to read to improve their growth mindsets. In study 1, high school students, undergraduates, and graduates were assigned to read either five struggle stories or five achievement stories of role models. Their mindsets were measured before and after reading the stories. The results showed that reading struggle stories rather than achievement stories of role models increased the growth mindsets of undergraduates and graduates. In study 2, undergraduates with high and low perseverance were assigned to read five struggle stories or five achievement stories of role models. Their mindsets were measured before reading stories and after reading each story. The results showed that the growth mindsets of undergraduates with low perseverance increased after reading two struggle stories of role models, and increased further after reading five struggle stories of role models. More importantly, the level of growth mindsets of undergraduates with low perseverance was equal to that of undergraduates with high perseverance after reading five struggle stories of role models. These findings reveal that reading struggle stories of role models can improve the growth mindsets of undergraduates and graduates. The personality of students affects the effectiveness of story-based mindset intervention.

## Introduction

Mindset is the belief of an individual about whether intelligence can be developed or not (Dweck and Leggett, 1988). Students who believe that intelligence cannot be changed are described as holding a “fixed mindset” (Dweck and Sorich, 1999). These students interpret setbacks as signs that they are not capable of succeeding; thus, they are lack motivation to work hard and give up easily after encountering setbacks (Dweck and Sorich, 1999; Blackwell et al., 2007). Conversely, students who believe that intelligence can be developed through effort are described as holding a “growth mindset” (Dweck and Sorich, 1999). These students have stronger motivation to work hard, display more persistent behavior even when faced with setbacks (Dweck and Sorich, 1999; Blackwell et al., 2007), and ultimately achieve academic success (Claro et al., 2016).

Mindset is a continuum that varies from a fixed mindset to a growth mindset (Dweck, 2006). Fixed mindsets of students increase with age. For instance, students in high school held stronger fixed mindsets than those in elementary school (Ablard and Mills, 1996) and middle school (Cheng and Hau, 2003). Students in high school and college held stronger fixed mindsets than those in elementary school (Gunderson et al., 2017). The increase of fixed mindsets may be due to the increase in the difficulty of courses. Previous studies found that the fixed mindsets of undergraduates in computer science (Flanigan et al., 2017), biology (Dai and Cromley, 2014), and math (Shively and Ryan, 2013) increased over the course of a semester. As the difficulty of courses in college is higher than that in high school, the fixed mindsets of undergraduates may be higher than that of high school students. Moreover, the fixed mindsets of teachers also influence the mindsets of students (de Ruiter et al., 2020; Davison et al., 2021). Scherr et al. (2017) paid attention to the fixed mindsets in physics graduate admissions and found that many teachers would choose students with a talent in physics so that they can be competent for more difficult graduate courses. Fixed mindsets of teachers may increase the fixed mindsets of graduates. Taken together, with the increase in the difficulty of courses and the fixed mindsets of teachers, the fixed mindsets held by high school students, undergraduates, and graduates are becoming very strong. Exploring intervention methods to improve the growth mindsets of high school students, undergraduates, and graduates can help them achieve academic success.

Stories about role models exemplify the successes of the role models in a specific field and provide behavioral templates that one can emulate to achieve success (Lockwood, 2006). When encountering setbacks, individuals usually get inspiration from stories about role models (McIntyre et al., 2011), changing their attitudes or beliefs, and motivating them to pursue their goals (Hong et al., 2015). Previous studies found that reading stories of women role models was beneficial for decreasing gender stereotypes of women (Dasgupta and Asgari, 2004; McIntyre et al., 2011) that reflect fixed mindsets. These findings suggest that reading stories of role models can improve the growth mindsets of individuals.

There are many achievement stories about role models (i.e., emphasizing achievements of role models without describing any setbacks) in textbooks, newspapers, and movies, and such stories have been widely used by educators to increase efforts or persistence of students in the face of academic setbacks. However, Hong and Lin-Siegler (2012) proposed that achievement stories of role models convey information that role models are innately talented individuals and their achievements depend on their natural gift. Students who read such stories believe that they do not have the talent to succeed, and intelligence cannot be changed (Hong and Lin-Siegler, 2012); thus, their growth mindsets decrease. In contrast, struggle stories of role models describe the role models as persistent individuals who make great efforts, overcome a series of setbacks, and ultimately achieve success (Hong and Lin-Siegler, 2012). Such stories highlight that intelligence can be developed through efforts, which can increase the growth mindsets of readers. Based on these perspectives, the current study aimed to clarify whether reading struggle stories of role models can improve the growth mindsets of students more effectively than reading achievement stories of role models.

Previous studies have successfully increased the growth mindsets of students by using a single-session intervention that includes reading struggle stories of role models, reading materials about the ability of the brain to grow, and so on (Yeager et al., 2016, 2019; Schleider and Weisz, 2017; Lewis et al., 2020). However, the effect of reading struggle stories of role models on growth mindsets is confused with the effect of other materials in the previous studies mentioned above. It is unclear whether only reading struggle stories of role models can solely increase the growth mindsets of students.

Other studies had compared the effects of reading struggle stories of role models or not on growth mindsets. The results found that reading struggle stories of role models increased the growth mindsets of college students (Shin et al., 2016) and improved the academic performance of college students (McIntyre et al., 2003; Shin et al., 2016) and adults (Herrmann et al., 2016). However, these studies did not compare the effect of reading struggle stories and achievement stories of role models on growth mindsets.

Only a small number of studies have compared the effects of reading struggle stories and achievement stories of role models on growth mindsets and academic performance. For instance, in Hong and Lin-Siegler (2012), high school students were assigned to the struggle story group (i.e., read three struggle stories of role models), the achievement story group (i.e., read three achievement stories of role models), or the control group (i.e., did not read the stories) in a 1-week intervention (read three times and read one story each time). The results showed that high school students in the struggle story group held more accurate images of scientists (i.e., as individuals who worked hard and overcame setbacks to achieve success) and had better academic performance than those in the achievement story group and the control group. It may suggest that reading struggle stories of role models can improve the growth mindsets of students better than reading achievement stories of role models.

However, in another study, ninth and tenth graders were assigned to the intellectual struggle story group (i.e., read three intellectual struggle stories of role models), the life struggle story group (i.e., read three life struggle stories of role models), or the achievement story group (i.e., read three achievement stories of role models) in a 3-week intervention (read one story each week). The results found that the participants in the two struggle story groups had better academic performance than those in the achievement story group, but there were no significant differences in growth mindsets among the three story groups (Lin-Siegler et al., 2016). It is unclear whether reading struggle stories of role models can improve the growth mindsets of students more effectively than reading achievement stories of role models.

It is noteworthy that Hong and Lin-Siegler (2012) used a 1-week intervention (read three times and one story each time) and suggested that reading struggle stories of role models improved the growth mindsets of high school students. Lin-Siegler et al. (2016) used a 3-week intervention (read one story each week) and found no significant differences in growth mindsets among the intellectual struggle story group, the life struggle story group, and the achievement story group. It may suggest that the interval between reading struggle stories of role models is important for the effectiveness of the intervention. Therefore, a 25-min single-session intervention (i.e., reading stories continually) was used in the current study to compare the effects of reading struggle stories and achievement stories of role models on growth mindsets. Moreover, previous studies mainly focused on middle school students (Hong and Lin-Siegler, 2012; Lin-Siegler et al., 2016), and whether reading struggle stories or achievement stories of role models can improve the growth mindsets of undergraduates and graduates needs to be further clarified. Thus, the first goal of the present study is to clarify whether the effect of reading struggle stories of role models on the growth mindsets of students is more effective than that of reading achievement stories of role models:

H1. Reading struggle stories of role models rather than achievement stories of role models increases the growth mindsets of high school students, undergraduates, and graduates.

Since mindset theory is a theory about the responses of individuals to setbacks (Yeager and Dweck, 2020), the personalities of individuals in the face of setbacks may affect the effect of reading specific stories about role models on the growth mindsets. However, to our knowledge, only one study focused on the impact of personality traits on the effectiveness of mindset intervention and found that mindset intervention was more effective among low grit students compared to high grit students (Orosz et al., 2017). Perseverance refers to the personality state in the face of setbacks (Bai et al., 2020). After experiencing setbacks, students with high perseverance have a proper understanding of the setbacks through introspection, regard the setbacks as controllable or changeable events, believe that they will achieve success ultimately, and face the setbacks without immersing in negative emotions (Bai et al., 2020). These students may have higher learning interests and better academic performance. In contrast, students with low perseverance perceive setbacks as uncontrollable or unchangeable events, are not confident they will succeed, are immersed in negative emotions, and give up easily (Bai et al., 2020). These students may have lower learning interests and poor academic performance. Previous studies found that reading three struggle stories of role models improved the learning interest of students with low learning interest but did not inspire the learning interest of students with high learning interest (Hong and Lin-Siegler, 2012), and improved academic performance of students with low academic performance but did not improve the academic performance of students with high academic performance (Lin-Siegler et al., 2016). Based on Bai et al. (2020), the findings mentioned above may suggest that mindset intervention is more effective for students with low perseverance. Thus, the second goal of the present study is to investigate how personality (i.e., perseverance) affects the effectiveness of mindset intervention and the number of struggle stories of role models undergraduates with low perseverance that need to be read to increase their growth mindsets.

H2. Story-based mindset intervention is more effective for undergraduates with low perseverance.
H3. Fewer struggle stories of role models are needed to increase the growth mindsets of undergraduates with low perseverance relative to those with high perseverance.

In summary, two studies were designed. In study 1, high school students, undergraduates, and graduates were asked to read five struggle stories or five achievement stories of role models. Their mindsets were measured before and after reading stories. In study 2, undergraduates with high and low perseverance were asked to read five struggle stories or five achievement stories of role models. Their mindsets were measured before reading stories and after reading each story.

## Study 1

### Methods

#### Participants

In total, 89 high school students, 90 undergraduates, and 81 graduates were randomly assigned to the achievement story group or the struggle story group. The number, gender, age, grade, and major of each group are presented in Table 1. A sample size calculation was done using the GPower 3.0 software, with a moderate size effect (*f* = 0.25), a power of 0.8 and an alpha error of 5%. The resulting sample size was 211 individuals. The sample size of study 1 met the requirements. Informed consent was obtained from the participants, and the study was approved by the Research Ethics Board of our university.

**Table 1:** The number, gender, age, grade, and major of each group.

**Variable**	**Struggle story group**			**Achievement story group**		
	**High school students**	**Undergraduates**	**Graduates**	**High school students**	**Undergraduates**	**Graduates**
**Gender**
Male	21	18	22	24	25	17
Female	24	26	19	20	21	23
**Age**	16.15 (1.08)	20.12 (1.72)	23.98 (1.28)	16.98 (0.53)	20.70 (0.95)	24.37 (1.44)
**Grade**
One	24	12	15	23	10	14
Two	15	17	16	15	15	18
Three	6	8	10	6	12	8
Four	–	7	–	–	9	–
**Major**
Liberal arts	–	30	32	–	33	27
Science(s)	–	14	9	–	13	13

#### Materials and Procedure

##### Mindset

The scale of beliefs about intelligence was developed by Blackwell et al. (2007). It contains three items reflecting that intelligence cannot be changed (fixed mindset) and three items reflecting that intelligence can be changed (growth mindset). Three fixed mindset items have been proven to be a good tool testing whether the mindset intervention successfully increased the growth mindsets (Yeager et al., 2013; Schleider and Weisz, 2016; Schleider et al., 2019a,b). Thus, three fixed mindset items were used in the current study to test whether reading specific stories increased the growth mindsets. A sample statement included “You have a certain amount of intelligence, and you really can't do much to change it.” The participants were asked to respond to each item on a 6-point scale ranging from 1 (strongly disagree) to 6 (strongly agree). The total score ranged from 3 to 18; higher scores represent a stronger fixed mindset, and lower scores represent a stronger growth mindset. Cronbach's alpha of three fixed mindset items was 0.86 for the pretest (*n* = 260).

The participants were asked to report their mindsets before reading the stories. The pretest mindset scores of the groups were compared. A 3 (grade: high school student, undergraduate, graduate) × 2 (story type: achievement story, struggle story) ANOVA was conducted. No main effects or interaction effects were found (*ps* > 0.05). The participants were asked to report their mindsets again after reading stories.

##### Stories of Role Models

Considering the influences of similarity between role models and readers on role models' effectiveness (Hu et al., 2020), famous Chinese physicists (Qian Xuesen, Deng Jiaxian), mathematicians (Chen Jingrun, Hua Luogeng), therapists (Zhong Nanshan, Tu Youyou), and biologists (Yuan Longping) were selected to make up stories.

According to previous studies (Hong and Lin-Siegler, 2012; Lin-Siegler et al., 2016), a struggle story and an achievement story were compiled for each scientist in the current study. Each story was ~500 words in length. The story types were reflected in the title and content of the stories. Struggle stories were titled “Great scientists have experienced setbacks: The story about × × ,” while achievement stories were titled “The Story of a Great Scientist: × × .”

All stories had a similar structure. The first paragraph of struggle and achievement stories introduced the important contributions of the scientists. The second and third paragraphs of the struggle stories introduced the work scientists were engaged in and their emotional experiences, beliefs, efforts, and ultimate outcomes after they encounter setbacks. The last paragraph of the struggle stories summarized that the successes of scientists depend on their efforts. In contrast, the second and third paragraphs of the achievement stories introduced the scientists' important discoveries and awards without describing any setbacks. The last paragraph of the achievement stories summarized the important influence of the scientists.

Twenty high school students (10 men and 10 women, *M*age = 16.11, *SD* = 0.52), 20 undergraduates (10 men and 10 women, *M*age = 19.20, *SD* = 0.84), and 20 graduates (10 men and 10 women, *M*age = 22.43, *SD* = 1.12) who did not participate in the formal experiment were asked to rate all stories. First, they were asked to rate their familiarity with each scientist on a 10-point scale (1 = very unfamiliar, 10 = very familiar) before reading the stories. Stories of two mathematicians whose familiarity scores were lower than chance level were excluded.

Second, according to Hong and Lin-Siegler (2012), achievement stories of role models should describe the role models as smart individuals who are born with a specific gift and their successes depend on the talent, while struggle stories of role models should describe the role models as individuals who make great efforts and their successes depend on their hard work. Thus, the participants were asked to rate whether each story convey the message correctly by answering a question on a 10-point scale (1 = talent, 10 = effort) before reading the stories (i.e., whether successes of individuals depend on talent or effort) and after reading each story (i.e., whether the successes of × × × depend on his/her talent or effort). The order of the stories was counterbalanced. The results showed that scores of the participants of the question after reading each struggle story were higher than those before reading the stories (*ps* < 0.05), whereas scores of the participants of the question after reading each achievement story were lower than (Deng Jiaxian, *p* < 0.05) or equal to (*ps* > 0.05) those before reading the stories. Moreover, for the same scientist, scores of the participants of the question after reading the achievement story were lower than those after reading the struggle story (*ps* < 0.05). It suggests that all stories convey the message correctly.

In the formal experiment, there were five struggle stories and five achievement stories. The order of the stories was counterbalanced. Participants were asked to rate their familiarity with each scientist on a 10-point scale (1 = very unfamiliar, 10 = very familiar) before reading the stories.

The brief introductions of the five scientists are as follows:

Qian Xuesen is the world's leading scientist in aerodynamics and the founder of manned space of China, An academician of the Chinese academy of sciences and the Chinese academy of engineering. He is also known as “the father of China's space.”

Tu Youyou discovered artemisinin, which is a drug therapy for malaria that has saved millions of lives across the globe, especially in the developing world. She received the 2015 Nobel Prize in Physiology or Medicine.

Deng Jiaxian is a leading organizer of the development of nuclear weapons in China. He successfully designed the atomic bomb and hydrogen bomb of China and led the development of self-defense weapons in China to an advanced level in the world.

Yuan Longping is a Chinese agronomist, known for developing the first hybrid rice. Hybrid rice has since been grown in dozens of countries in Africa, America, and Asia, and acts as a robust food source in areas with a high risk of famine.

Zhong Nanshan is a famous respiratory expert. He was the hero who defeated SARS in 2003 and was awarded the Medal of the Republic for his contributions toward fighting against COVID-19.

#### Experimental Design

A 3 (grade: high school student, undergraduate, graduate) × 2 (story type: achievement story, struggle story) experimental design was conducted. The grade and the story type were between-subject variables. The dependent variable was post-test mindset scores.

#### Statistical Analysis

A *z*-test was applied for the normality test using skewness and kurtosis (Kim, 2013). The tests of normality and homogeneity of data were compared, and an analysis of covariance (ANCOVA) was conducted on this basis in SPSS 23.0.

### Results

In order to clarify whether the effect of reading struggle stories of role models on growth mindsets of high school students, undergraduates, and graduates is more effective than that of reading achievement stories of role models, an analysis of covariance (ANCOVA) was conducted with grade and story type as the independent variables, post-test mindset scores as the dependent variable, and familiarity scores and pretest mindset scores as the covariates.

A main effect of the grade was found, *F*_(2, 252)_ = 9.73, *p* < 0.001, η^2^ = 0.072. The mindset scores of the high school students (*M* = 10.07, *SD* = 3.23) were significantly higher than those of the undergraduates (*M* = 8.97, *SD* = 3.66, *p* < 0.001) and graduates (*M* = 8.67, *SD* = 3.80, *p* = 0.002). There was no significant difference in mindset scores between undergraduates and graduates (*p* = 1.000). The results reveal that high school students hold stronger fixed mindsets than undergraduates and graduates.

A main effect of the story type was found, *F*_(1, 252)_ = 63.91, *p* < 0.001, η^2^ = 0.202. The mindset scores of the participants after reading struggle stories of role models (*M* = 7.84, *SD* = 3.33) were significantly lower than those after reading achievement stories of role models (*M* = 10.66, *SD* = 3.30). The results reveal that students who read struggle stories of role models hold stronger growth mindsets than those who read achievement stories of role models.

**Figure 1:**
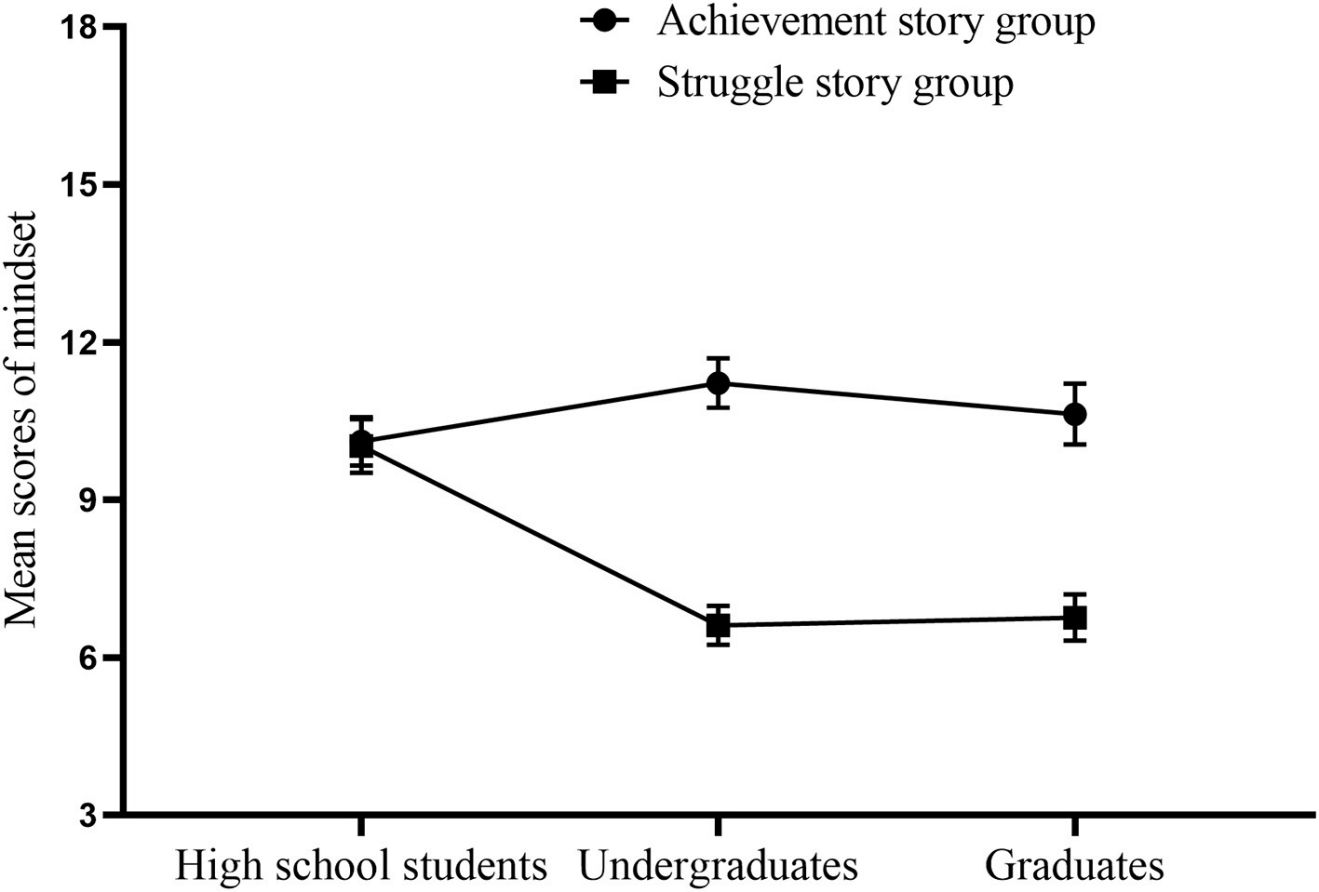
The mindset scores of high school students, undergraduates, and graduates after reading struggle or achievement stories. Error bars represent standard error.

A significant interaction was found between the story type and the grade, *F*_(2, 252)_ = 16.04, *p* < 0.001, η^2^ = 0.113 (see Figure 1). Simple effect analysis found that there was no main effect of the grade in the achievement story group, *F*_(2, 252)_ = 0.43, *p* = 0.653. The main effect of the grade was found in the struggle story group, *F*_(2, 252)_ = 25.50, *p* < 0.001, η^2^ = 0.168. The mindset scores of the high school students were higher than those of the undergraduates (*p* < 0.001) and graduates (*p* < 0.001) after reading struggle stories of role models. There was no significant difference in mindset scores between the undergraduates and graduates (*p* = 1.000) after reading struggle stories of role models. Moreover, the mindset scores of the undergraduates (*p* < 0.001) and graduates (*p* < 0.001) after reading struggle stories of role models were lower than those after reading achievement stories of role models. There was no significant difference in mindset scores between the high school students in the achievement story group and those in the struggle story group (*p* = 0.899). These findings reveal that reading struggle stories of role models can increase the growth mindsets of undergraduates and graduates.

## Study 2

### Methods

#### Participants

In total, 210 undergraduates underwent the undergraduate perseverance scale (Bai et al., 2020). According to Lü et al. (2016), participants who scored one standard deviation (SD) above mean on the undergraduate perseverance scale (i.e., high-perseverance group), and those who scored one SD below the mean on the undergraduate perseverance scale (i.e., low-perseverance group) were selected to participate in the experiment. The sample consisted of 80 participants. They were randomly assigned to the struggle story group or the achievement story group. The number, gender, age, grade, and major of each group are presented in Table 2. A sample size calculation was done using the GPower 3.0 software with a moderate size effect (*f* = 0.25), a power of 0.8, and an alpha error of 0.05. The resulting sample size was 36 individuals. The sample size of study 2 met the requirements. All participants signed prior informed consent before the experiment. The experimental processes were approved by the ethics committee of our university.

**Table 2:** The number, gender, age, grade, and major of each group.

**Variable**	**Struggle story group**		**Achievement story group**	
	**High perseverance**	**Low perseverance**	**High perseverance**	**Low perseverance**
**Gender**
Male	11	8	8	10
Female	8	12	13	10
**Age**	20.18 (1.79)	20.05 (1.95)	20.23 (1.74)	20.30 (1.61)
**Grade**
One	7	8	6	5
Two	5	7	9	6
Three	4	2	3	6
Four	3	3	3	3
**Major**
Liberal arts	11	14	15	12
Science(s)	8	6	6	8

#### Materials and Procedure

##### Mindset

We used the same items as in study 1. The participants were asked to report their mindsets before reading the stories. The pretest mindset scores of the groups were compared. A 2 (perseverance group: high, low) × 2 (story type: achievement story, struggle story) ANOVA was conducted. Only the main effect of perseverance group on mindset scores were found, *F*_(1, 76)_ = 31.59, *p* < 0.001, η^2^ = 0.294. The participants with high perseverance held stronger growth mindsets than those with low perseverance. The pretest mindset scores served as the baseline level (i.e., reading zero stories). Cronbach's alpha of three fixed mindset items was 0.91 for the pretest (*n* = 80). The participants were asked to report their mindsets after reading each story.

##### Perseverance

The undergraduate perseverance scale (Bai et al., 2020) measures the perseverance of undergraduates. Of 20 items in total, eight items measure conviction (e.g., “Although I encountered setbacks, the previous achievements make me believe that my goals will be achieved eventually”), four items measure controllability (e.g., “Setbacks are because of bad luck”), three items measure enlightenment (e.g., “If I keep working hard, I will achieve good results after setbacks”), and five items measure optimism (e.g., “I feel spiritless on anything after I encounter setbacks”). Participants needed to respond on a 6-point scale ranging from 0 (strongly disagree) to 5 (strongly agree). The Cronbach's alpha for the total scale is 0.89 (Bai et al., 2020). In the present study, Cronbach's alpha was 0.86 (*n* = 210).

##### Stories of Role Models

We used the same materials and procedure as in study 1.

#### Experimental Design

A 2 (perseverance group: high, low) × 2 (story type: achievement story, struggle story) × 6 (number of stories: 0, 1, 2, 3, 4, 5) mixed experimental design was conducted. The perseverance group and the story type were between-subject variables, while the number of stories was a within-subject variable. The dependent variable was mindset scores.

#### Statistical Analysis

A z-test was applied for the normality test using skewness and kurtosis (Kim, 2013). The tests of normality and homogeneity of data were met, on which an analysis of covariance (ANCOVA) under a mixed model was conducted in SPSS 23.0. Mauchly's test of sphericity yielded an alpha level <0.05, and the degrees of freedom were adjusted using the Greenhouse–Geisser correction.

### Results

In order to clarify the effect of reading struggle stories of role models on growth mindsets of undergraduates with high and low perseverance, and the number of struggle stories of role models, undergraduates with low perseverance needs to read to increase their growth mindsets; an ANCOVA under mixed model was conducted with perseverance, story type, and number of stories as the independent variables mindset scores as the dependent variable, and familiarity scores, age and major as the covariates.

A main effect of the story type was found, *F*_(1, 73)_ = 19.79, *p* < 0.001, η^2^ = 0.213. The mindset scores of the participants after reading struggle stories of role models (*M* = 7.91, *SD* = 3.00) were significantly lower than those after reading achievement stories of role models (*M* = 10.37, *SD* = 3.45). The results reveal that undergraduates who read struggle stories of role models hold stronger growth mindsets than those who read achievement stories of role models.

A main effect of the perseverance was found, *F*_(1, 73)_ = 26.05, *p* < 0.001, η^2^ = 0.263. The mindset scores of the participants with low perseverance (*M* = 10.89, *SD* = 2.92) were significantly higher than those of the participants with high perseverance (*M* = 7.45, *SD* = 3.08). The results reveal that undergraduates with high perseverance hold stronger growth mindsets than undergraduates with low perseverance.

A significant interaction between the story type and the number of stories was found, *F*_(2.43, 177.38)_ = 41.12, *p* < 0.001, η^2^ = 0.360. Simple effect analysis found a main effect of the number of stories in the achievement story group, *F*_(5, 69)_ = 4.19, *p* = 0.002, η^2^ = 0.233. The mindset scores of the participants after reading zero (*M* = 9.85, *SD* = 3.50), one (*M* = 9.90, *SD* = 3.40), two (*M* = 10.02, *SD* = 3.46) stories were significantly lower than those after reading four (*M* = 10.93, *SD* = 3.76) and five (*M* = 11.15, *SD* = 4.00) stories (*ps* < 0.05). The mindset scores of the participants after reading three stories (*M* = 10.34, *SD* = 3.55) were significantly lower than those after reading four stories (*p* = 0.021). A main effect of the number of stories in the struggle story group was found, *F*_(5, 69)_ = 15.63, *p* < 0.001, η^2^ = 0.531. The mindset scores of the participants after reading zero stories (*M* = 9.41, *SD* = 4.04) were significantly higher than those after reading one (*M* = 8.87, *SD* = 3.47), two (*M* = 8.08, *SD* = 3.17), three (*M* = 7.62, *SD* = 2.93), four (*M* = 7.28, *SD* = 2.78), and five (*M* = 6.23, *SD* = 2.57) stories (*ps* < 0.05). The mindset scores of the participants after reading one story were significantly higher than those after reading two, three, four, and five stories (*ps* < 0.001). The mindset scores of the participants after reading two stories were significantly higher than those after reading four and five stories (*ps* < 0.05). The mindset scores of the participants after reading three and four stories were significantly higher than those after reading five stories (*ps* < 0.05). The results reveal that the growth mindsets of the undergraduates decrease after reading four achievement stories of role models, while increase after reading one struggle story of a role model.

**Figure 2:**
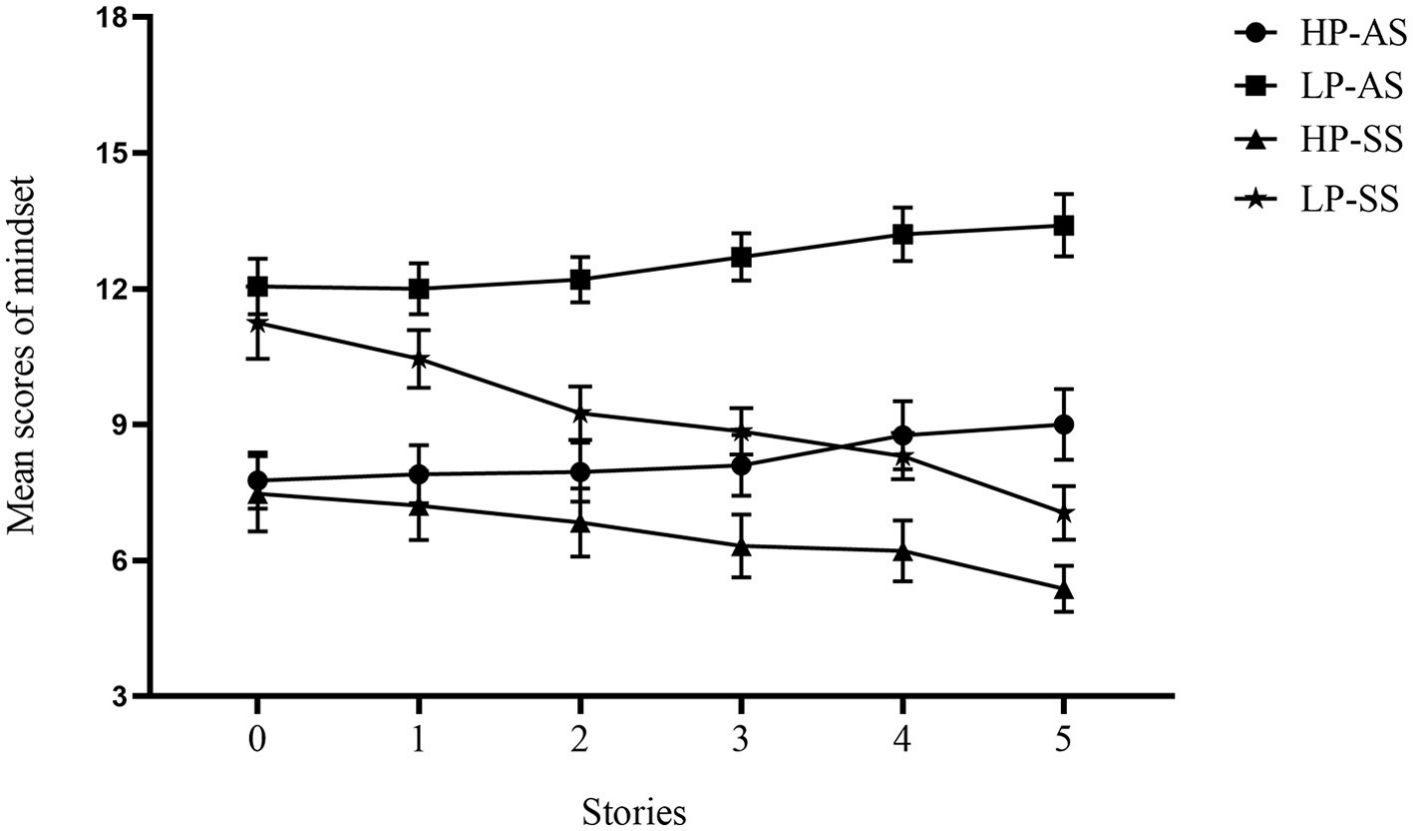
The mindset scores of undergraduates with high and low perseverance after reading five struggle or five achievement stories. Error bars represent standard error. Number of stories: the number of stories participants have read. HP-AS group: a high-perseverance group that read achievement stories of role models. LP-AS group: a low-perseverance group that read achievement stories of role models. HP-SS group: a high-perseverance group that read struggle stories of role models. LP-SS group: a low-perseverance group that read struggle stories of role models.

A significant interaction among the story type, perseverance, and number of stories was found, *F*_(2.43, 177.38)_ = 3.21, *p* = 0.033, η^2^ = 0.042 (see Figure 2).

For the undergraduates with low perseverance in the struggle story group, their mindset scores after reading zero and one struggle stories of role models were higher than those after reading two, three, four, and five struggle stories of role models (*ps* < 0.05). The mindset scores after reading two, three, and four struggle stories of role models were higher than those after reading five struggle stories of role models (*ps* < 0.05). For the undergraduates with high perseverance in the struggle story group, their mindset scores after reading zero struggle stories of role models were higher than those after reading two, three, four, and five struggle stories of role models (*ps* < 0.05). The mindset scores after reading the one struggle story of a role model were higher than those after reading three, four, and five struggle stories of role models (*ps* < 0.05). The mindset scores after reading two struggle stories of role models were higher than those after reading five struggle stories of role models (*p* = 0.021). The results reveal that the growth mindsets of undergraduates with high and low perseverance increase after reading two struggle stories of role models and increase further after reading five struggle stories of role models.

For the undergraduates with low perseverance, their mindset scores after reading two struggle stories were lower than those after reading two achievement stories of role models (*p* = 0.002). Their mindset scores after reading three struggle stories were lower than those after reading three achievement stories of role models (*p* < 0.001). Their mindset scores after reading four struggle stories were lower than those after reading four achievement stories of role models (*p* < 0.001). Their mindset scores after reading five struggle stories were lower than those after reading five achievement stories of role models (*p* < 0.001). For the undergraduates with high perseverance, their mindset scores after reading four struggle stories were lower than those after reading four achievement stories of role models (*p* = 0.010). Their mindset scores after reading five struggle stories were lower than those after reading five achievement stories of role models (*p* < 0.001). The results reveal that undergraduates with low perseverance begin to hold stronger growth mindsets after reading two struggle stories of role models, compared to reading two achievement stories of role models. In contrast, undergraduates with high perseverance begin to hold stronger growth mindsets after reading four struggle stories of role models, compared with reading four achievement stories of role models.

For the struggle story group, the mindset scores in undergraduates with high perseverance were lower than those in undergraduates with low perseverance after reading zero, one, two, three, and four struggle stories of role models (*ps* < 0.05). There was no significant difference between the mindset scores in undergraduates with high perseverance and those in undergraduates with low perseverance after reading five struggle stories of role models (*p* = 0.133). For the achievement story group, the mindset scores in undergraduates with high perseverance were lower than those in undergraduates with low perseverance after reading zero, one, two, three, four, and five achievement stories of role models (*ps* < 0.001). The results reveal that the level of growth mindsets of undergraduates with low perseverance is equal to that of undergraduates with high perseverance after reading five struggle stories of role models.

Other effects were not found (*ps* > 0.05).

## General Discussion

The current study examined whether reading struggle stories of role models can improve the growth mindsets of students and the number of struggle stories of role models undergraduates with high and low perseverance need to read to improve their mindsets. The results showed that reading struggle stories rather than achievement stories of role models can improve the growth mindsets of undergraduates and graduates. The growth mindsets of undergraduates with high and low perseverance improved after reading two struggle stories of role models and improved further after reading five struggle stories of role models. More importantly, the level of growth mindsets of undergraduates with low perseverance was equal to that of undergraduates with high perseverance after reading five struggle stories of role models. Moreover, undergraduates with low perseverance began to hold stronger growth mindsets after reading two struggle stories of role models, compared with reading two achievement stories of role models, while undergraduates with high perseverance began to hold stronger growth mindsets after reading four struggle stories of role models, compared with reading four achievement stories of role models.

As is reflected in our hypothesis, study 1 found that reading struggle stories of role models increased the growth mindsets of undergraduates and graduates, which is consistent with the findings of previous studies (McIntyre et al., 2003; Herrmann et al., 2016). As mentioned above, struggle stories of role models convey information that success depends on efforts and intelligence can be developed (Hong and Lin-Siegler, 2012), which increases the growth mindsets of readers. Moreover, reading struggle stories of role models can make readers believe that “role models, like us, need to overcome setbacks before they succeed.” This belief helps to enhance the similarity between the role models and readers, and thus the readers are more likely to be influenced by role models (Hu et al., 2020).

Study 1 found that reading struggle stories of role models did not increase the growth mindsets of high school students, which is inconsistent with our hypothesis. Hong and Lin-Siegler (2012) found that high school students in the struggle story group perceived scientists as more hard-working individuals than those in the achievement story group. However, the perception of role models as hard-working individuals may be only the first step to improve the growth mindsets of high school students. The key element that induced belief change is inducing dissatisfaction with one's original beliefs (Grube et al., 1994). High school students are facing the pressures from college entrance examinations and are more likely to experience more academic setbacks. Their original mindsets may be more stable and are more difficult to change than those of undergraduates and graduates. They may need to read more struggle stories of role models to change their growth mindsets (Lin-Siegler et al., 2016).

Study 2 found that the growth mindsets of undergraduates with high and low perseverance improved after reading two struggle stories of role models. Female undergraduates received a message that women have no talent for math (i.e., reflecting a fixed mindset) and were then asked to read zero, one, two, three, or four struggle stories of female role models, respectively (McIntyre et al., 2005). The results showed that reading the one struggle story of a female role model increased the beliefs of female undergraduates about the ability of women to do well in math relative to reading zero stories. However, McIntyre et al. (2005) only focused on the effect of reading struggle stories of female role models on female undergraduates, and their conclusions cannot extend to male participants.

Consistent with our hypothesis, study 2 found that undergraduates with low perseverance began to hold stronger growth mindsets after reading two struggle stories of role models, compared with reading two achievement stories of role models, while undergraduates with high perseverance began to hold stronger growth mindsets after reading four struggle stories of role models, compared with reading four achievement stories of role models. These findings reveal that story-based mindset intervention is more effective for students with low perseverance, which is consistent with the findings of previous studies (Hong and Lin-Siegler, 2012; Lin-Siegler et al., 2016).

Moreover, study 2 found that the level of growth mindsets in undergraduates with low perseverance was equal to that in undergraduates with high perseverance after reading five struggle stories of role models. The result reveals the effectiveness of story-based mindset intervention for undergraduates with low perseverance. Future studies should investigate how many struggle stories of role models undergraduates with low perseverance need to be read to improve their growth mindsets to surpass those of undergraduates with high perseverance.

The present study has significant implications for the field of mindset intervention studies. First, the current study clarifies that reading struggle stories of role models can improve growth mindsets, while reading achievement stories of role models cannot, which confirms the perspectives of Hong and Lin-Siegler (2012). Second, the current study found that reading struggle stories of role models can improve the growth mindsets of undergraduates and graduates but cannot improve those of high school students. This finding reveals the applicable scope of struggle story-based mindset intervention. Third, the present study examines the number of struggle stories of role models, undergraduates with high and low perseverance needs to read to improve their growth mindsets, which further reveals the effect of personality factors on story-based mindset intervention.

The current study also has some limitations. It uses the method of an oral presentation to investigate the growth mindset. Recent studies have focused on behavioral indicators of a growth mindset, such as challenge-seeking behavior (Yeager et al., 2016, 2019). Future research should adopt multiple dependent variables of mindset to investigate the effect of reading struggle stories of role models on growth mindset.

In conclusion, the current study reveals that reading struggle stories of role models is an effective tool to improve the growth mindsets of undergraduates and graduates. Personality influences the effectiveness of story-based mindset intervention.

## Data Availability Statement

The datasets presented in this study can be found in online repositories. The names of the repository/repositories and accession number(s) can be found below: The datasets [study 1 data] for this study can be found at this link: [https://figshare.com/s/5b058858c30aba4aa9e5]. The datasets [study 2 data] for this study can be found at this link [https://figshare.com/s/f4644a4ba75db7ca8a1c]. User name: duxu1987@163.com password: Duxu_121314.

## Ethics Statement

The studies involving human participants were reviewed and approved by the Research Ethics Board of the Tianjin Normal University. Written informed consent to participate in this study was provided by the participants' legal guardian/next of kin.

## Author Contributions

XD and XB contributed to the conception of the study. XD wrote the draft of the manuscript and designed the study. SY and YL performed the statistical analysis. All authors contributed to the article and approved the submitted version.

## Funding

This work was supported by the National Social Science Foundation of China (Grant No. 20ZDA079).

## Conflict of Interest

The authors declare that the research was conducted in the absence of any commercial or financial relationships that could be construed as a potential conflict of interest.

## Publisher's Note

All claims expressed in this article are solely those of the authors and do not necessarily represent those of their affiliated organizations, or those of the publisher, the editors and the reviewers. Any product that may be evaluated in this article, or claim that may be made by its manufacturer, is not guaranteed or endorsed by the publisher.

## References

[B1] Ablard K. E. Mills C. J. (1996). Implicit theories of intelligence and self-perceptions of academically talented adolescents and children. J. Youth Adolesc. 25, 137–148. 10.1007/BF01537340

[B2] Bai X. Du X. Niu H. Hao J. (2020). The exploration of the structure of perseverance: Based on the measurement of undergraduates. Stud. Psychol. Behav. 18, 638–644. 10.3969/j.issn.1672-0628.2020.05.010

[B3] Blackwell L. S. Trzesniewski K. H. Dweck C. S. (2007). Implicit theories of intelligence predict achievement across an adolescent transition: a longitudinal study and an intervention. Child Dev. 78, 246–263. 10.1111/j.1467-8624.2007.00995.x 17328703

[B4] Cheng Z. J. Hau K. T. (2003). Are intelligence and personality changeable? Generality of Chinese students' beliefs across various personal attributes and age groups. Pers. Individ. Diff. 34, 731–748. 10.1016/S0191-8869(02)00030-2

[B5] Claro S. Paunesku D. Dweck C. S. (2016). Growth mindset tempers the effects of poverty on academic achievement. Proc. Natl. Acad. Sci. U. S. A. 113:8664. 10.1073/pnas.1608207113 27432947 PMC4978255

[B6] Dai T. Cromley J. G. (2014). Changes in implicit theories of ability in biology and dropout from stem majors: a latent growth curve approach. Contemp. Educ. Psychol. 39, 233–247. 10.1016/j.cedpsych.2014.06.003

[B7] Dasgupta N. Asgari S. (2004). Seeing is believing: exposure to counterstereotypic leaders and its effects on the malleability of automatic gender stereotyping. J. Exp. Soc. Psychol. 40, 642–658. 10.1016/j.jesp.2004.02.003

[B8] Davison D. P. Wijnen F. M. Charisi V. Meij J. Evers V. (2021). Words of encouragement: how praise delivered by a social robot changes children's mindset for learning. J. Multimodal User Interfaces. 15, 61–76. 10.1007/s12193-020-00353-9

[B9] de Ruiter N. M. P. van der Klooster K. N. Thomaes S. (2020). “Doing” mindsets in the classroom: a coding scheme for teacher and student mindset-related verbalizations. J. Person Oriented Res. 6, 103–119. 10.17505/jpor.2020.22404 33569155 PMC7871171

[B10] Dweck C. S. (2006). Mindset: The New Psychology of Success. New York, NY: Random House.

[B11] Dweck C. S. Leggett E. L. (1988). A social cognitive approach to motivation and personality. Psychol. Rev. 95, 256–273. 10.1037/0033-295X.95.2.256

[B12] Dweck C. S. Sorich L. A. (1999). “Mastery-oriented thinking,” in Coping, ed. C. R. Snyder (Oxford: Oxford University Press), 232–251.

[B13] Flanigan A. E. Peteranetz M. S. Shell D. A. Soh L. K. (2017). Implicit intelligence beliefs of computer science students: exploring change across the semester. Contemp. Educ. Psychol. 48, 179–196. 10.1016/j.cedpsych.2016.10.003

[B14] Grube J. W. Mayton D. M. Ball-Rokeach S. J. (1994). Inducing change in values, attitudes, and behaviors: belief system theory and the method of value self-confrontation. J. Soc. Issues 50, 153–173. 10.1111/j.1540-4560.1994.tb01202.x

[B15] Gunderson E. A. Hamdan N. Sorhagen N. S. D'Esterre A. P. (2017). Who needs innate ability to succeed in math and literacy? Academic-domain-specific theories of intelligence about peers versus adults. Dev. Psychol. 53, 1188–1205. 10.1037/dev0000282 28383932

[B16] Herrmann S. D. Adelman R. M. Bodford J. E. Graudejus O. Okun M. A. Kwan V. S. Y. (2016). The effects of a female role model on academic performance and persistence of women in stem courses. Basic Appl. Soc. Psychol. 38, 258–268. 10.1080/01973533.2016.1209757

[B17] Hong H. Lin-Siegler X. (2012). How learning about scientists' struggles influences students' interest and learning in physics. J. Educ. Psychol. 104, 469–484. 10.1037/a0026224

[B18] Hong H. Y. Chai C. S. Tsai C. C. (2015). College students constructing collective knowledge of natural science history in a collaborative knowledge building community. J. Sci. Educ. Technol. 24, 549–561. 10.1007/s10956-015-9546-8

[B19] Hu D. Ahn J. N. Vega M. Lin-Siegler X. (2020). Not all scientists are equal: role aspirants influence role modeling outcomes in STEM. Basic Appl. Soc. Psychol. 42, 192–208. 10.1080/01973533.2020.1734006

[B20] Kim H. Y. (2013). Statistical notes for clinical researchers: Assessing normal distribution (2) using skewness and kurtosis. Restorative Dentistry Endodontics 38, 52–54. 10.5395/rde.2013.38.1.52 23495371 PMC3591587

[B21] Lewis L. S. Williams C. A. Dawson S. D. (2020). Growth mindset training and effective learning strategies in community college registered nursing students. Teach. Learn. Nurs. 15, 123–127. 10.1016/j.teln.2020.01.006

[B22] Lin-Siegler X. Ahn J. N. Chen J. Fang F. A. LunaLucero M. (2016). Even Einstein struggled: effects of learning about great scientists' struggles on high school students' motivation to learn science. J. Educ. Psychol. 108, 314–328. 10.1037/edu0000092

[B23] Lockwood P. (2006). “Someone like me can be successful”: do college students need same-gender role models? Psychol. Women Quart. 30, 36–46. 10.1111/j.1471-6402.2006.00260.x

[B24] Lü W. Wang Z. You X. (2016). Physiological responses to repeated stress in individuals with high and low trait resilience. Biol. Psychol. 120, 46–52. 10.1016/j.biopsycho.2016.08.005 27543044

[B25] McIntyre R. B. Lord C. G. Gresky D. M. Ten Eck L. L. Frye J. G. D. Bond C. F. (2005). A social impact trend in the effects of role models on alleviating women's mathematics stereotype threat. Curr. Res. Soc. Psychol. 10, 116–136. Available online at: https://foothill.edu/attach/1474/Role_Models.pdf

[B26] McIntyre R. B. Paulson R. M. Lord C. G. (2003). Alleviating women's mathematics stereotype threat through salience of group achievements. J. Exp. Soc. Psychol. 39, 83–90. 10.1016/S0022-1031(02)00513-9

[B27] McIntyre R. B. Paulson R. M. Taylor C. A. Morin A. L. Lord C. G. (2011). Effects of role model deservingness on overcoming performance deficits induced by stereotype threat. Eur. J. Soc. Psychol. 41, 301–311. 10.1002/ejsp.774

[B28] Orosz G. Péter-Szarka S. Bothe B. Tóth-Király I. Berger R. (2017). How not to do a mindset intervention: learning from a mindset intervention among students with good grades. Front. Psychol. 8:311. 10.3389/fpsyg.2017.00311 28337158 PMC5343031

[B29] Scherr R. E. Plisch M. Gray K. E. Potvin G. Hodapp T. (2017). Fixed and growth mindsets in physics graduate admissions. Phys. Rev. Phys. Educ. Res. 13:020133. 10.1103/PhysRevPhysEducRes.13.020133

[B30] Schleider J. L. Burnette J. L. Widman L. Hoyt C. Prinstein M. J. (2019a). Randomized trial of a single-session growth mind-set intervention for rural adolescents' internalizing and externalizing problems. J. Clin. Child Adolesc. Psychol. 49, 660–672. 10.1080/15374416.2019.1622123 31219698 PMC6923626

[B31] Schleider J. L. Mullarkey M. C. Weisz J. (2019b). Virtual reality and web-based growth mindset interventions for adolescent depression: protocol for a three-arm randomized trial. JMIR Res. Protoc. 8:e13368. 10.2196/13368 31290406 PMC6647760

[B32] Schleider J. L. Weisz J. R. (2016). Reducing risk for anxiety and depression in adolescents: effects of a single-session intervention teaching that personality can change. Behav. Res. Ther. 87, 170–181. 10.1016/j.brat.2016.09.011 27697671 PMC5127737

[B33] Schleider J. L. Weisz J. R. (2017). A single-session growth mindset intervention for adolescent anxiety and depression: 9-month outcomes of a randomized trial. J. Child Psychol. Psychiatry Allied Disciplines 59, 160–170. 10.1111/jcpp.12811 28921523

[B34] Shin J. E. L. Levy S. R. London B. (2016). Effects of role model exposure on STEM and non-STEM student engagement. J. Appl. Soc. Psychol. 46, 410–427. 10.1111/jasp.12371

[B35] Shively R. L. Ryan C. S. (2013). Longitudinal changes in college math students' implicit theories of intelligence. Soc. Psychol. Educ. 16, 241–256. 10.1007/s11218-012-9208-0

[B36] Yeager D. S. Dweck C. S. (2020). What can be learned from growth mindset controversies? Am. Psychol. 75, 1269–1284. 10.1037/amp0000794 33382294 PMC8299535

[B37] Yeager D. S. Hanselman P. Walton G. M. Murray J. S. Crosnoe R. Muller C.. (2019). A national experiment reveals where a growth mindset improves achievement. Nature 573, 364–369. 10.1038/s41586-019-1466-y 31391586 PMC6786290

[B38] Yeager D. S. Romero C. Paunesku D. Hulleman C. S. Schneider B. Hinojosa C.. (2016). Using design thinking to improve psychological interventions: the case of the growth mindset during the transition to high school. J. Educ. Psychol. 108, 374–391. 10.1037/edu0000098 27524832 PMC4981081

[B39] Yeager D. S. Trzesniewski K. H. Dweck C. S. (2013). An implicit theories of personality intervention reduces adolescent aggression in response to victimization and exclusion. Child Dev. 84, 970–988. 10.1111/cdev.12003 23106262 PMC3660787

